# How comparable are patient outcomes in the “real-world” with populations studied in pivotal AML trials?

**DOI:** 10.1038/s41408-024-00996-x

**Published:** 2024-03-26

**Authors:** Ing Soo Tiong, Meaghan Wall, Ashish Bajel, Akash Kalro, Shaun Fleming, Andrew W. Roberts, Nisha Thiagarajah, Chong Chyn Chua, Maya Latimer, David Yeung, Paula Marlton, Amanda Johnston, Anoop Enjeti, Chun Yew Fong, Gavin Cull, Stephen Larsen, Glen Kennedy, Anthony Schwarer, David Kipp, Sundra Ramanathan, Emma Verner, Campbell Tiley, Edward Morris, Uwe Hahn, John Moore, John Taper, Duncan Purtill, Pauline Warburton, William Stevenson, Nicholas Murphy, Peter Tan, Ashanka Beligaswatte, Howard Mutsando, Mark Hertzberg, Jake Shortt, Ferenc Szabo, Karin Dunne, Andrew H. Wei, Australasian Leukaemia and Lymphoma Group (ALLG)

**Affiliations:** 1 Peter MacCallum Cancer Centre, Melbourne, VIC Australia; 2 The Alfred Hospital, Melbourne, VIC Australia; 3 Monash University, Melbourne, VIC Australia; 4 Victorian Clinical Genetics Services, Murdoch Children’s Research Institute, Melbourne, VIC Australia; 5 Royal Melbourne Hospital, Parkville, VIC Australia; 6 The University of Melbourne, Melbourne, VIC Australia; 7 Royal Adelaide Hospital, Adelaide, SA Australia; 8 Walter and Eliza Hall Institute of Medical Research, Melbourne, VIC Australia; 9 The Northern Hospital, Epping, VIC Australia; 10 Canberra Hospital, Garran, ACT Australia; 11 ACT Pathology, Garran, ACT Australia; 12 Australian National University, Canberra, ACT Australia; 13 South Australian Health and Medical Research Institute, Adelaide, SA Australia; 14 Princess Alexandra Hospital, Woolloongabba, QLD Australia; 15 University of Queensland, Brisbane, QLD Australia; 16 Westmead Hospital, Westmead, NSW Australia; 17 Calvary Mater Newcastle, Waratah, NSW Australia; 18 Austin Hospital, Heidelberg, VIC Australia; 19 Sir Charles Gairdner Hospital, Nedlands, WA Australia; 20 PathWest Laboratory Medicine, Nedlands, WA Australia; 21 University of Western Australia, Perth, WA Australia; 22 Royal Prince Alfred Hospital, Camperdown, NSW Australia; 23 Royal Brisbane and Women’s Hospital, Herston, QLD Australia; 24 Box Hill Hospital, Box Hill, VIC Australia; 25 Barwon Health, Geelong, VIC Australia; 26 St George Hospital, Kogarah, NSW Australia; 27 Concord Hospital, Concord, NSW Australia; 28 Gosford Hospital, Gosford, NSW Australia; 29 University of Newcastle, Callaghan, NSW Australia; 30 Townsville University Hospital, Douglas, QLD Australia; 31 The Queen Elizabeth Hospital, Woodville South, SA Australia; 32 SA Pathology, Adelaide, SA Australia; 33 St Vincent’s Hospital Sydney, Darlinghurst, NSW Australia; 34 Nepean Hospital, Kingswood, NSW Australia; 35 Fiona Stanley Hospital, Murdoch, WA Australia; 36 Wollongong Hospital, Wollongong, NSW Australia; 37 Royal North Shore Hospital, St Leonards, NSW Australia; 38 Northern Clinical School, University of Sydney, Sydney, NSW Australia; 39 Royal Hobart Hospital, Hobart, TAS Australia; 40 Royal Perth Hospital, Perth, WA Australia; 41 Flinders Medical Centre, Bedford Park, SA Australia; 42 Flinders University, Bedford Park, SA Australia; 43 Toowoomba Hospital, Toowoomba, QLD Australia; 44 Prince of Wales Hospital, Randwick, NSW Australia; 45 Monash Medical Centre, Clayton, VIC Australia; 46 Royal Darwin Hospital, Tiwi, NT Australia; 47 Australasian Leukaemia and Lymphoma Group (ALLG), Melbourne, VIC Australia

**Keywords:** Acute myeloid leukaemia, Epidemiology, Acute myeloid leukaemia

## Abstract

Despite an increasing desire to use historical cohorts as “synthetic” controls for new drug evaluation, limited data exist regarding the comparability of real-world outcomes to those in clinical trials. Governmental cancer data often lacks details on treatment, response, and molecular characterization of disease sub-groups. The Australasian Leukaemia and Lymphoma Group National Blood Cancer Registry (ALLG NBCR) includes source information on morphology, cytogenetics, flow cytometry, and molecular features linked to treatment received (including transplantation), response to treatment, relapse, and survival outcome. Using data from 942 AML patients enrolled between 2012–2018, we assessed age and disease-matched control and interventional populations from published randomized trials that led to the registration of midostaurin, gemtuzumab ozogamicin, CPX-351, oral azacitidine, and venetoclax. Our analyses highlight important differences in real-world outcomes compared to clinical trial populations, including variations in anthracycline type, cytarabine intensity and scheduling during consolidation, and the frequency of allogeneic hematopoietic cell transplantation in first remission. Although real-world outcomes were comparable to some published studies, notable differences were apparent in others. If historical datasets were used to assess the impact of novel therapies, this work underscores the need to assess diverse datasets to enable geographic differences in treatment outcomes to be accounted for.

## Introduction

One concern regarding the control arm of clinical trial populations, owing to strict patient selection criteria, is their generalizability to real-world AML outcomes. In 2012 the cooperative trial group, Australasian Leukaemia and Lymphoma Group (ALLG), established the National Blood Cancer Registry (NBCR) to serve as an umbrella registration framework for patients with acute myeloid leukemia (AML) intended for enrolment to ALLG clinical trials or standard of care (SOC) therapy, and link the data to the ALLG biobank. Participant recruitment to the ALLG NBCR is ongoing and covers the majority of hospitals providing active anti-leukemic therapy for patients with AML in Australia. The ALLG NBCR functions as a web-based interface, permitting entry of patient and AML disease characteristics, type and dose of chemotherapy received, transplantation details, clinical response and overall survival outcomes. The major strength of the ALLG NBCR is the collection of de-identified source data enabling verifiable analysis of full blood examination, bone marrow morphology, flow cytometric, cytogenetic and molecular parameters at diagnosis and at each response assessment, with primary reports scanned and stored into an electronic database. Using source data reports, the AML diagnosis for each patient is confirmed within the ALLG NBCR by an independent hematologist, and cytogenetic risk verified by an expert cytogeneticist.

A series of recent drug approvals has expanded the range of first-line therapeutic options available to patients with AML [1]. Randomized studies have demonstrated improved outcomes over SOC with addition of midostaurin to patients with *FLT3* mutant AML [2], gemtuzumab ozogamicin (GO) for de novo CD33 + AML [3, 4], venetoclax for patients ineligible for intensive chemotherapy [5, 6], ivosidenib for elderly, unfit patients with *IDH1* mutated AML [7], CPX-351 for patients with secondary/therapy-related AML [8] and oral azacitidine as maintenance therapy for patients ineligible for allogeneic hematopoietic cell transplantation (HCT) (Table 1) [9]. Concerningly, wide variation in AML clinical practice already existed prior to the introduction of these recently approved new drugs.

**Table 1:** Summary of key clinical outcomes in trials leading to recent approvals for AML therapies.

Trial Therapy	Regulatory approval in the US and Australia	Target population	SOC therapy	CR/CRi rates (%)		Median OS (months)	
				Active	Control	Active	Control
Midostaurin*	FDA: 28-Apr-2017 TGA: 17-May-2018 PBS: 01-Dec-2018	*FLT3* mutant AML Age 18–59 y	7 + 3	58.9	53.5	74.7	25.6
Gemtuzumab ozogamicin†	FDA: 01-Sep-2017 TGA: 09-Apr-2020 PBS: 01-Mar-2022	De novo AML Age 50–70 y	7 + 3	81.3	74.8	27.5	21.8
CPX-351	FDA: 03-Aug-2017 TGA: 03-Jun-2022 PBS: n/a	Secondary or therapy related AML Age 60–75 y	7 + 3	47.7	33.3	9.6	6.0
CC-486	FDA: 01-Sep-2020 TGA: 08-Apr-2022 PBS: 01-Sep-2023	AML in CR1 and not candidates for HCT Age ≥55 y	Nil	-	-	24.7	14.8
Venetoclax^§^	FDA: 21-Nov-2018 TGA: 20-Apr-2020 PBS: 01-Dec-2021	Age ≥75 y or unfit	AZA	66.4	28.3	14.7	9.6
Venetoclax^§^	FDA: 21-Nov-2018 TGA: n/a PBS: n/a	Age ≥75 y or unfit	LDAC	48	13	8.4	4.1
Ivosidenib	FDA: 25-May-2022 TGA: 06-Apr-2023 PBS: n/a	Age ≥75 y or unfit	AZA	54	16	24.0	7.9

*Included only CR achieved by 60 days.

^†^Included CR and CRp; median OS as per updated analysis (Lambert, *Haematologica* 2019).

^§^Venetoclax in combination with azacitidine, or decitabine, or LDAC was granted accelerated approval by FDA on 21-Nov-2018, followed by full approval on 16-Oct-2020. In Australia, the approval is only for use in combination with azacitidine.

*AML* acute myeloid leukemia, *AZA* azacitidine, *CR* complete remission, *CR1* first remission, *CRi* CR with incomplete hematologic recovery, *CRp* CR with incomplete platelet recovery, *HCT* hematopoietic cell transplantation, *LDAC* low-dose cytarabine, *n/a* not available, *OS* overall survival, *PBS* Pharmaceutical Benefits Scheme, *SOC* standard of care, *TGA* Therapeutic Goods Administration, *y* years.

To illustrate similarities and differences in what was considered “standard of care” in the control arm of clinical AML studies used to support recent drug approvals by the FDA with current SOC in Australia, we undertook a comparative analysis with defined patient sub-populations in the NBCR matched to the control arm of published randomized studies involving frontline AML drugs. Real-world data comprised information from patients enrolled to the ALLG NBCR between 2012 and 2018, prior to reimbursed access to new AML drugs in Australia.

## Subjects and methods

The ALLG NBCR (ACTRN12612000337875) was approved by the Human Research and Ethics Committees of the Alfred Hospital (181/12) and Royal Melbourne Hospital (2012.105) and activated in December 2012. Written informed patient consent was obtained from all participating patients. Standardized web-based electronic data case report forms collected details of patient demography, AML diagnosis, including baseline diagnostic laboratory assessment, treatment received, disease status and survival outcome (Table S1). At registration, patients were invited to consent to storage of diagnostic bone marrow and/or blood samples to a tissue repository for future research. An expert cytogeneticist was appointed to centrally review de-identified cytogenetic reports to confirm karyotypic risk classification using Medical Research Council (MRC) 2010 criteria [10]. To enable comparison with published studies, cytogenetic risk was reclassified to align with the relevant study being assessed, including European LeukemiaNet (ELN) 2010 [11] or National Comprehensive Cancer Network (NCCN) recommendations. Molecular testing was performed according to local standard practice. A panel of 4 hematologists centrally reviewed bone marrow, flow cytometric, cytogenetic and molecular reports to assign patients to an AML World Health Organization (WHO) 2008 diagnostic category, as this was the classification schema used in most of the AML studies examined [12]. Serial follow-up was conducted to monitor for disease and survival status.

For the current analysis, a data extract was performed on 20-Aug-2020 for patients registered up to 30-April-2018 from 35 treatment centers. Continuous variables were expressed as medians or means according to the clinical trial in comparison. Categorical variables were summarized as counts and percentages. Group comparisons were performed using the Fisher’s exact test. Overall survival (OS) was calculated from date of diagnosis or, if missing, date of registration to date of death from any cause. Relapse-free survival was from the date of first remission to date of relapse or death from any cause. Kaplan–Meier survival curves were compared using log-rank statistics. All tests were two-sided and considered significant where *p* < 0.05. Statistical analyses by R statistical software version 3.6.2.

## Results

### Recruitment summary

Between December 2012 and April 2018, the ALLG NBCR registered 1022 patients (Fig. S1). Patients with acute promyelocytic leukemia (*n* = 62) were excluded from analyses. Another 18 patients were excluded because of a diagnosis other than AML, leaving a total of 942 patients in the final analysis cohort (Fig. S2). During this period, 11% of NBCR participants were also co-enrolled to an ALLG clinical trial, including the AMLM15 (*n* = 20; ACTRN12610000627055), AMLM16 (*n* = 81; ACTRN12611001112954) and AMLM21 (*n* = 1; ACTRN12614000810617) studies (Supplementary Information).

### Data coverage and quality

Data quality for relevant data fields are summarized in Table S2. Baseline bone marrow morphology, flow cytometry and cytogenetic reports were available for most patients (>90%). Molecular pathology results for *FLT3*-ITD, *NPM1*, *IDH1/*2 and *CEBPA* were available in 82%, 69%, 20% and 14%, respectively. Details regarding first line treatment were available for approximately 90% of the population. Median follow up for surviving patients was 32 months (range <1 to 107 months). For deceased patients, cause of death was provided in 89% of instances.

### Baseline characteristics

Patient demographic and disease characteristics are summarized in Table S3. The initial phase of the clinical registry focused on enrolment of patients fit for intensive chemotherapy (median age 57, range 16–80), received by 85% of patients, with allogeneic HCT performed in 21% of the study population. Low-intensity therapy (median age 75, range 54–92) and best supportive care (median age 75, range 57–88) were delivered to 9% and 4% patients in the registry, respectively. The entire study population had a slight male preponderance (55%). The median baseline white blood count (WBC) was 7.4 × 10^9^/L, with 8% of patients presenting with hyperleukocytosis (WBC > 100 × 10^9^/L). The proportion of patients diagnosed with de novo AML was 88%, whereas 12% of cases had AML with a prior history of cytotoxic therapy or radiotherapy (5.4%), myelodysplastic syndrome (MDS, 4.2%), chronic myelomonocytic leukemia (CMML, 1.8%) or myeloproliferative neoplasm (MPN, 0.7%). Cytogenetic data was available in 92% patients: with favorable, intermediate, adverse or unknown cytogenetic risk in 10%, 63%, 19% and 8%, respectively. Complex (≥3 abnormalities) and monosomal karyotype were present in 16% and 12% of patients. The commonest AML sub-groups were AML with myelodysplasia-related changes (AML-MRC; 28.3%) and AML not otherwise specified (28.0%). Mutations were detected in *NPM1* (35%), *FLT3*-ITD (23%), *IDH1* (14.5%), *IDH2* (16.8%), *CEBPA* (6.7% biallelic) and *FLT3*-TKD (6%).

### Registry-based outcomes for patients with *FLT3* mutant AML aged 18–59 years prior to the introduction of midostaurin

Reimbursed midostaurin became available on the Australian Pharmaceutical Benefits Scheme (PBS) from December 2018, with approval based on the pivotal RATIFY trial in which 707 patients aged 18–59 years with newly diagnosed *FLT3* mutation positive AML were randomized (between 2008 and 2011) to either midostaurin or placebo in combination with intensive induction and consolidation therapy. Addition of midostaurin improved 4-year OS (51% vs 44%), the primary endpoint of the study [2]. The 60-day complete remission (CR) rate was similar in the midostaurin and placebo arms (59% vs 54%).

**Fig. 1:**
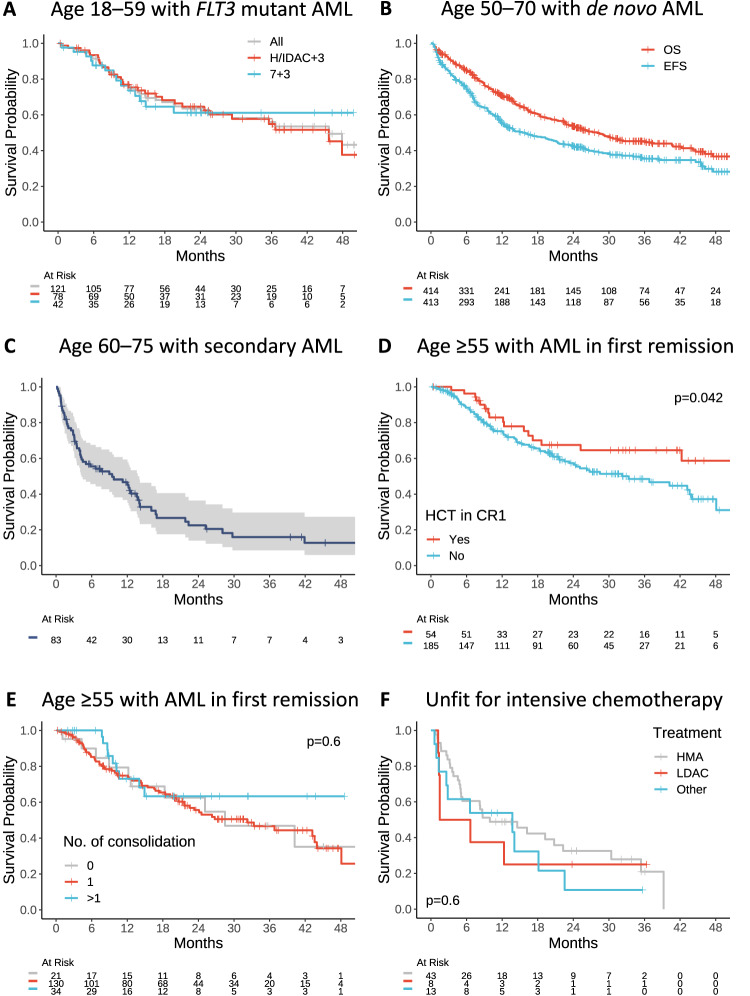
Kaplan Meier survival curves among the key subgroups of ALLG NBCR cohort. Log-rank statistics are used for comparisons. **A** Overall survival (OS) among all patients aged 18–59 years with *FLT3*-ITD and TKD mutated AML who received intensive chemotherapy, and according to the induction intensity (high or intermediate-dose cytarabine with anthracycline [H/IDAC + 3] versus standard 7 + 3). Missing induction regimen data for 1 patient. **B** OS and event-free survival (EFS) among patients aged 50–70 years with de novo AML who received intensive chemotherapy. Missing OS and EFS outcome data for 4 and 5 patients, respectively. **C** OS among patients aged 60–75 years with secondary or therapy-related AML who received intensive chemotherapy. Missing data for 2 patients. (**D** + **E**) OS (with 4-month landmark) among patients aged ≥55 years in first remission (CR1) according to (**D**) allogeneic HCT in CR1, and (**E**) number of consolidation chemotherapy received among patients (*n* = 185) not proceeding to HCT in CR1. **F** OS among patients who were unfit for intensive chemotherapy, according to the low-intensity regimens received.

In the ALLG NBCR cohort, 121 patients aged 18–59 years with newly diagnosed *FLT3*-ITD (allelic ratio ≥0.05; *n* = 103) and *FLT3*-TKD (*n* = 19; 1 had concurrent ITD) mutated AML treated with intensive chemotherapy without midostaurin were identified. The *FLT3*-ITD allelic ratio was <0.5, 0.5–0.7 and >0.7 in 43%, 16% and 24% of patients, respectively. *FLT3*-TKD was mutated in 20% of patients. Compared to the RATIFY study, patients in the ALLG NBCR were similar in terms of median age, gender distribution, *FLT3*-ITD allelic ratio and frequency of concurrent *NPM1* mutation (Table 2). The major baseline difference was a higher median WBC at diagnosis in the ALLG NBCR cohort (42 × 10^9^/L), compared to the RATIFY study population (33–36 × 10^9^/L), and more patients had favorable cytogenetic risk (9% vs 5–6%). Induction in the ALLG NBCR cohort utilized idarubicin as the main anthracycline as part of a 7 + 3 (35%) or high-dose cytarabine-based (65%) induction regimen, with both induction approaches delivering similar OS outcomes in *FLT3* mutated AML (Fig. 1A).

**Table 2:** Characteristics and outcomes of patients enrolled to the RATIFY study or the ALLG NBCR aged 18–59 with *FLT3* mutation receiving intensive chemotherapy^#^.

Characteristics	RATIFY – EXP (*n* = 360)	RATIFY – PBO (*N* = 357)	NBCR (*N* = 121)
Age, years, median (range)	47.1 (19.0–59.8)	48.6 (18.0–60.9)	44 (18–59)
Male gender (%)	48.3	40.6	47.9
White blood cell (x10^9^/L), median (range)	35.6 (0.6–421.8)	33.0 (0.8–329.8)	42.4 (1.3–349.2)
Cytogenetic risk according to ELN 2010, *n* (%)*			
- Favorable	16/269 (5.9)	13/278 (4.7)	10/113 (8.8)
- Intermediate	231/269 (85.9)	248/278 (89.2)	100/113 (88.5)
- Adverse	22/269 (8.2)	17/278 (6.1)	3/113 (2.7)
*FLT3* mutation subtype, *n* (%)			
- TKD	81 (22.5)	81 (22.7)	19/93 (20.4)
- ITD allelic ratio <0.5	171 (47.5)	170 (47.6)	52 (43.0)
- ITD allelic ratio 0.5–0.7			19 (15.7)
- ITD allelic ratio >0.7	108 (30.0)	106 (29.7)	32 (26.4)
*NPM1* mutation, *n* (%)†	244/427 (57.1)		57/101 (56.4)
Treatment response, *n* (%)			
- CR	212 (58.9)	191 (54.0)	70/116 (60.3)
- Death	16 (4.4)	11 (3.1)	2/116 (1.7)
Allogeneic HCT, *n* (%)			
- All patients	213 (59.2)	196 (54.9)	60 (49.6)
- In first remission	101 (28.1)	81 (22.7)	56 (46.3)
OS, months, median (95% CI)	74.7 (31.5–NR)	25.6 (18.6–42.9)	45.7 (29.3–NR)
OS at 4 years, % (95% CI)‡	51.4	44.3	43.2 (29.8–62.7)

^#^*N* represents the total population; for individual variables, some data were not measured/reported, and the appropriate denominator is specified in these instances. Some percentages do not add up to 100 due to rounding.

*Modified to combine both normal karyotype and intermediate II categories into a single intermediate group.

^†^Data on *NPM1* mutation were not separately reported for treatment arms (Döhner et al. Blood 2020).

^‡^95% CI not reported by the RATIFY study. Note median follow-up was 59 and 25 months in the RATIFY study and the NBCR cohort, respectively.

*CR* complete remission, *CI* confidence interval, *ELN* European LeukemiaNet, *EXP* experimental, *HCT* hematopoietic cell transplantation, *ITD* internal tandem duplication, *NR* not reached, *OS* overall survival, *PBO* placebo, *TKD* tyrosine kinase domain.

Rates of CR in the ALLG NBCR cohort and the control arm of RATIFY were similar (60% vs 54%) (Table 2). Although overall rates of HCT were also similar, more patients underwent HCT in first remission in the ALLG NBCR cohort, compared to the RATIFY study (46 vs 25%, *p* < 0.001). With a median follow-up time of 25 months for patients in the ALLG NBCR cohort, the median OS was 45.7 months (95% CI, 29.3–NR) (Fig. 1A), compared to 25.6 months (95% CI, 18.6–42.9) and 74.7 months (95% CI, 31.5–NR) for patients in the placebo or midostaurin arms of the RATIFY study, respectively. The estimated 4-year OS was 43.2% (95% CI, 29.8–62.7) in the NBCR cohort, compared to 44.3% and 51.4% for patients in the placebo or midostaurin arms of the RATIFY study, respectively.

### Registry-based outcomes for patients with de novo AML aged 50–70 years prior to the introduction of gemtuzumab ozogamicin

The French ALFA-0701 study randomized 280 patients to intensive chemotherapy (7 days of infusional cytarabine plus 3 days of daunorubicin) with or without GO 3 mg/m^2^ on days 1, 4, and 7 during induction and day 1 of each of 2 consolidation courses. The study enrolled patients with de novo AML aged 50–70 years between 2008–2010. GO was associated with an improved 2-year event free survival (EFS) (40.8% vs 17.1%), the primary endpoint of the study [3, 4].

GO was publicly subsidized by the PBS in Australia from March 2022. In the ALLG NBCR, 523 patients were aged between 50–70 years and 447 had de novo AML, of which 418 (94%) received intensive induction chemotherapy without GO. Table 3 compares the characteristics and outcomes of patients enrolled to the ALLG NBCR and both arms of the ALFA-0701 study. Baseline median age, cytogenetic and molecular characteristics were similar between the two cohorts. In the ALLG NBCR cohort, idarubicin (12 mg/m^2^) was the dominant anthracycline (93%) used in induction, compared to daunorubicin (60 mg/m^2^) in the ALFA-0701 study. The ALFA-0701 trial utilized daunorubicin in combination with intermediate-dose cytarabine (1 g/m^2^) as the consolidation backbone. Consolidation regimens in the ALLG NBCR cohort included intermediate-dose (1–1.9 g/m^2^ in 39%) or high-dose (≥2 g/m^2^ in 25%) cytarabine, with only 30% utilizing anthracycline. The ALLG NBCR cohort had a higher rate of allogeneic HCT in first remission (21% vs 14% compared to the ALFA-0701 study, *p* = 0.03).

**Table 3:** Characteristics and outcomes of patients enrolled to the ALFA-0701 study or the ALLG NBCR aged 50–70 years with de novo AML receiving induction chemotherapy^#^.

Characteristics	ALFA – EXP (*N* = 139)	ALFA – PBO (*N* = 139)	NBCR (*N* = 418)
Age, years, median (IQR)	62.8 (59.3, 66.8)	61.7 (57.4, 65.6)	61.0 (56.0, 66.0)
- Age ≥60, *n* (%)	100 (72)	86 (62)	246 (59)
Male gender, *n* (%)	77 (55)	61 (44)	235 (56)
White blood cell (x10^9^/L), median (IQR)	6.9 (2.3, 30.4)	5.0 (1.9, 26.7)	5.2 (2.1, 21.4)
Platelet count (x10^9^/L), median (IQR)	66.0 (36.5, 118.5)	67.5 (36.3, 125.5)	65.0 (38.0, 127.5)
Cytogenetic risk according to study definition, n (%)			
- Favorable	3/122 (2.5)	6/127 (4.7)	36/397 (9.1)
- Intermediate	91/122 (74.6)	91/127 (71.7)	274/397 (69.0)
- Adverse	28/122 (23.0)	30/127 (23.6)	87/397 (21.9)
*FLT3*-ITD, *n* (%)	22/137 (16.1)	27/138 (19.6)	76/366 (20.8)
*NPM1* mutation, *n* (%)	45/136 (33.1)	48/138 (34.8)	113/307 (36.8)
Favorable ELN 2010, *n* (%)	24/119 (20.2)	24/125 (19.2)	61/359 (17.0)
Induction courses >1	25 (18.0)	35 (25.2)	42 (10.0)
Treatment response, *n* (%)*			
- CR	102 (73.4)	100 (71.9)	259/396 (65.4)
- CRp	11 (7.9)	4 (2.9)	57/396 (14.4)
- No CR/CRp	17 (12)	29 (21)	67/396 (16.9)
- Death	9 (6.4)	6 (4.3)	13/396 (3.3)
Allogeneic HCT, *n* (%)			
- All patients	32 (23.7)	53 (39.0)	109 (26.1)
- In first remission	17/135 (12.6)	22 (16.2)	87/417 (20.9)
EFS, months, median (95% CI)	15.6 (11.7–22.4)	9.7 (8.0–11.9)	15.9 (12.3–21.4)
EFS at 2 years, % (95% CI)	40.8 (32.8–50.8)	17.1 (10.8–27.1)	42.7 (37.8–48.2)
OS, months, median (95% CI)^†^	27.5 (21.4–45.6)	21.8 (15.5–27.4)	28.1 (22.7–40.6)
OS at 2 years, % (95% CI)	53.2 (44.6–63.5)	41.9 (33.1–53.1)	53.5 (48.4–59.1)

^#^*N* represents the total population; for individual variables, some data were not measured/reported, and the appropriate denominator is specified in these instances. Some percentages do not add up to 100 due to rounding.

*CRp rates are not available for the NBCR; CRi rates are reported instead.

^†^Median OS for the ALFA-0701 cohort was based on the study update analysis (Lambert, *Haematologica* 2019).

*CR* complete remission, *CRp* CR with incomplete platelet recovery, *EFS* event-free survival, *ELN* European LeukemiaNet, *EXP* experimental, *HCT* hematopoietic cell transplantation, *IQR* interquartile range, *NCCN* National Comprehensive Cancer Network, *OS* overall survival, *PBO* placebo.

For patients recorded as receiving intensive chemotherapy in the ALLG NBCR, the CR rate was 65% (vs 72% for the SOC [7 + 3] arm of ALFA-0701), 2-year EFS was 43% (95% CI, 38–48) (vs 17% in the SOC arm of ALFA [95% CI, 11–27]) and 2-year OS 54% (95% CI, 48–59) (vs 42% in the SOC arm of ALFA [95% CI, 33–53]) (Fig. 1B).

### Registry-based outcomes for patients with secondary/therapy-related AML aged 60–75 years prior to the introduction of CPX-351

CPX-351 was examined in an open label registration study, which randomized 309 patients between 2012–2014 to either 7 + 3 induction followed by 2 cycles of 5 + 2 consolidation, or CPX-351 for 1-2 induction cycles followed by consolidation with a similar regimen. The study enrolled patients with secondary or therapy-related AML aged 60–75 years. CPX-351 was associated with an improved median OS (9.6 vs 6.0 months), the primary endpoint of the study, and a higher complete response rate (48% vs 33%) [8]. The improved OS was maintained at 5-year follow-up: 18% vs 8% [13].

CPX-351 has been approved for marketing in Australia by the Therapeutic Goods Administration but is yet to be publicly subsidized in Australia. In the ALLG NBCR cohort, 387 patients were aged between 60–75 years and 85 (22%) received intensive chemotherapy for secondary (85%) or therapy-related (15%) AML. Secondary AML was defined by myelodysplasia-related cytogenetic abnormalities and/or prior MDS/CMML; AML-MRC based solely on morphology and those with antecedent myeloproliferative neoplasm were excluded from this analysis as per the eligibility criteria of the CPX-351 registration study [8].

Table 4 compares the characteristics and outcomes of patients enrolled to the CPX-351 study or the ALLG NBCR. Patients in the ALLG NBCR were less frequently ≥70 years (19% vs 36%, *p* = 0.003), more commonly had a baseline WBC ≥ 20 (31% vs 15%, *p* = 0.008) and more frequently had adverse risk karyotype (69% vs 50%, *p* = 0.02). The dominant anthracycline used for induction in the ALLG NBCR cohort was idarubicin (90%), compared to daunorubicin for patients in the control arm of the CPX-351 study. Furthermore, intermediate-dose cytarabine consolidation was used among 43% (15/35) of patients in the ALLG NBCR cohort, compared to 5 + 2 consolidation for all patients in the control arm of the CPX-351 study. Allogeneic HCT was more common in the CPX-351 study (29% vs 7% in the NBCR cohort, *p* < 0.001).

**Table 4:** Characteristics and outcomes of patients enrolled to the CPX-351 study or the ALLG NBCR aged 60–75 years with secondary or therapy-related AML receiving induction chemotherapy^#^.

Characteristics	CPX-351 (*N* = 153)	7 + 3 (*N* = 156)	NBCR (*N* = 85)
Age, years, mean (SD)	67.8 (4.2)	67.7 (4.1)	65.7 (3.8)
- Age 60–69, *n* (%)	96 (62.7)	102 (65.4)	69 (81.2)
- Age 70–75, *n* (%)	57 (37.3)	54 (34.6)	16 (18.8)
Male gender, *n* (%)	94 (61.4)	96 (61.5)	49 (57.6)
White blood cell, *n* (%)			
- <20 × 10^9^/L	131 (85.6)	131/155 (84.5)	59 (69.4)
- ≥20 × 10^9^/L	22 (14.4)	24/155 (15.5)	26 (30.6)
Cytogenetic risk according to NCCN version 2.2011, *n* (%)*			
- Favorable	7/143 (4.9)	5/146 (3.4)	1/81 (1.2)
- Intermediate	64/143 (44.8)	58/146 (39.7)	24/81 (29.6)
- Adverse	72/143 (50.3)	83/146 (56.8)	56/81 (69.1)
*FLT3* mutation, *n* (%)^†^	22/138 (15.9)	21/141 (14.9)	10/64 (15.6)
AML subtype, *n* (%)			
- Therapy-related AML	30 (19.6)	33 (21.2)	13 (15.3)
- AML with antecedent MDS	71 (46.4)	74 (47.4)	18 (21.2)
- AML with antecedent CMML	11 (7.2)	12 (7.7)	4 (4.7)
- De novo with MDS karyotype	41 (26.8)	37 (23.7)	50 (58.8)
Treatment response, *n* (%)			
- CR/CRi	47.7%	33.3%	42/79 (53.2)
- CR	37.3%	25.6%	28/79 (35.4)
- TRM at 30 days	5.9%	10.6%	11/83 (13.3)
- TRM at 60 days	13.7%	21.2%	20/83 (24.1)
Allogeneic HCT in CR1, *n* (%)	52 (34.0)	39 (25.0)	6 (7.1)
EFS, months, median (95% CI)	2.53 (2.07–4.99)	1.31 (1.08–1.64)	4.01 (2.43–7.06)
OS, months, median (95% CI)	9.56 (6.60–11.86)	5.95 (4.99–7.75)	9.46 (4.3–13.8)
OS landmarked from HCT, months, median (95% CI)	NR	10.25 (6.21–16.69)	11.07 (9.89–NR)

^#^*N* represents the total population; for individual variables, some data were not measured/reported, and the appropriate denominator is specified in these instances. Some percentages do not add up to 100 due to rounding.

*NCCN version was not specified in the original publication. As the patient enrollment commenced in 2012 in the CPX-351 study, we have classified the patients in the NBCR cohort using NCCN version 2.2011.

^†^*FLT3* ITD versus TKD status was not specified in the original publication. The NBCR cohort included 8 patients with *FLT3*-ITD and 2 patients with *FLT3*-TKD.

*AML* acute myeloid leukemia, *CMML* chronic myelomonocytic leukemia, *CR1* first remission, *CR* complete remission, *CRi* CR with incomplete hematologic recovery, *EFS* event-free survival, *HMA* hypomethylating agent, *HCT* hematopoietic cell transplantation, *MDS* myelodysplastic syndrome, *NCCN* National Comprehensive Cancer Network, *NR* not reached, *NR* not reached, *OS* overall survival, *SD* standard deviation, *TRM* treatment-related mortality.

Rates of CR/CRi in the ALLG NBCR cohort were 53% vs 33% (*p* = 0.005) in the control arm of the CPX-351 study. With a median follow-up time of 40 months in the ALLG NBCR cohort, median OS was 9.5 months (95% CI, 4.3–13.8) (Fig. 1C), compared to 5.95 months (95% CI, 4.99–7.75) in the control arm of the CPX-351 study.

### Registry-based outcomes for patients aged ≥ 55 years in first remission after intensive chemotherapy prior to the introduction of oral azacitidine

The QUAZAR study randomized 472 patients between 2013–2017 to either oral azacitidine maintenance therapy or placebo. The study enrolled patients with non-CBF AML ≥ 55 years in first CR or CR with incomplete hematologic recovery (CRi) after induction +/− consolidation therapy among patients considered ineligible for allogeneic HCT. The primary endpoint of the trial was OS. Oral azacitidine was associated with an improved median OS (25 vs 15 months). Median time from CR to randomization was 85 days [9].

Oral azacitidine was listed on the PBS in Australia on 01-Sep-2023. In the ALLG NBCR cohort, induction chemotherapy was administered to 406 patients ≥55 years with non-CBF AML. CR or CRi after one or two (*n* = 34) induction cycles was achieved in 278 (68%) patients. After excluding early relapses or deaths within 85 days, 239 patients were included in the analysis, among which 54 patients (23%) underwent allogeneic HCT in the first remission, compared to 14% in the no maintenance arm of the QUAZAR study. The remaining 185 patients in the ALLG NBCR cohort exhibited a profile similar to the study population enrolled in the QUAZAR study; characteristics and outcomes are summarized in Table 5. Median age in the ALLG NBCR and the QUAZAR study were comparable (65 vs 68 years) and a similar proportion had adverse cytogenetic risk (~15%) or de novo AML (94% vs 91%). The proportion of patients in QUAZAR receiving none, one or ≥2 consolidation cycles after achieving CR/CRi were 20%, 45% and 35%, respectively, compared to 11%, 70% and 18% of patients in the ALLG NBCR cohort (*p* < 0.001).

**Table 5:** Characteristics and outcomes of patients enrolled to the QUAZAR study or the ALLG NBCR aged ≥55 years in CR or CRi after intensive chemotherapy^#^.

Characteristics	CC-486 (EXP) (*N* = 238)	CC-486 (PBO) (*N* = 234)	NBCR (no HCT) (*N* = 185)	NBCR (HCT) (*N* = 54)	P*
Age, years, median (range)	68 (55–86)	68 (55–82)	65 (55–77)	59 (55–69)	<0.001
- Age ≥65 years, *n* (%)	172 (72)	166 (71)	94 (51)	10 (19)	<0.001
Male gender, *n* (%)	118 (50)	127 (54)	103 (56)	29 (54)	0.9
Cytogenetic risk according to NCCN version 2.2011, *n* (%)					0.04
- Intermediate	203 (85)	203 (87)	155/181 (86)	39 (72)	
- Adverse	35 (15)	31 (13)	26/181 (14)	15 (28)	
De novo AML, *n* (%)	213 (89)	216 (92)	174 (94)	49 (91)	0.4
Treatment response, *n* (%)					1
- CR	187 (79)	197 (84)	155 (84)	46 (85)	
- CRi	51 (21)	37 (16)	30 (16)	8 (15)	
Received consolidation, *n* (%)					0.4
- No	52 (22)	42 (18)	21 (11)	8 (15)	
- 1 cycle	110 (46)	102 (44)	130 (70)	40 (74)	
- ≥2 cycles	76 (32)	90 (39)	34 (18)	6 (11)	
OS, months, median (95% CI)	24.7 (18.7–30.5)	14.8 (11.7–17.6)	32.2 (23.3–48.1)	NR (42.3–NR)	0.04

^#^*N* represents the total population; for individual variables, some data were not measured/reported, and the appropriate denominator is specified in these instances. Some percentages do not add up to 100 due to rounding.

*Comparison between HCT or no HCT in first remission in the ALLG NBCR cohort. Student’s t-test for continuous variables, Fisher’s exact test for categorical variables, and log-rank test for overall survival analysis (4-month landmark).

*AML* acute myeloid leukemia, *CR* complete remission, *CRi* CR with incomplete hematologic recovery, *EXP* experimental, *HCT* hematopoietic cell transplantation, *NCCN* National Comprehensive Cancer Network, *NR* not reached, *OS* overall survival, *PBO* placebo.

With a median follow-up time of 30.7 months in the ALLG NBCR cohort (4-month landmark), the median OS for patients who did not proceed to allogeneic HCT was 32.2 months (vs not reached in those who underwent HCT in first remission) (Fig. 1D), compared to 14.8 and 24.7 months for patients in the QUAZAR study receiving placebo or CC-486, respectively (Table 5). Among the ALLG NBCR cohort not proceeding to allogeneic HCT in first remission (*n* = 185), there was no significant difference in OS outcome for patients receiving 0, 1 or ≥2 consolidation cycles (Fig. 1E).

### Registry-based outcomes for patients receiving non-intensive therapy prior to the introduction of venetoclax

Venetoclax in combination with azacitidine became available for general use in Australia from December 2021 for older patients unfit for intensive chemotherapy. The VIALE-A study enrolled patients between 2017–2019 with newly diagnosed AML ≥ 75 years or ineligible to receive intensive chemotherapy and excluded those with prior hypomethylating agent (HMA) exposure. In VIALE-A, 431 patients were randomized to either venetoclax or placebo in combination with azacitidine. The venetoclax arm had an improved median OS (14.7 vs 9.6 months) and rate of CR/CRi (66.4% vs 28.3%) over placebo [5].

The VIALE-C study also enrolled patients between 2017–2018 with newly diagnosed AML who were either ≥75 years or ineligible to receive intensive chemotherapy, but in contrast to VIALE-A, permitted patients with prior HMA exposure, a group which comprised 20% patients. A total of 211 patients were randomized to either venetoclax or placebo in combination with low-dose cytarabine (LDAC). In a *post-hoc* secondary analysis using more mature follow-up data, venetoclax was associated with an improved median OS (8.4 vs 4.1 months) and CR/CRi (48% vs 13%) [6].

In the ALLG NBCR, a total of 121 patients were deemed unfit and not given intensive chemotherapy. Seventeen patients received venetoclax and 40 patients opted for best supportive care only and were excluded from analysis. The remaining 64 patients received low-intensity therapy with a median age of 77 years, which was similar to VIALE-A (76 years) and VIALE-C (76 years) (Table 6). The proportion of patients with adverse cytogenetic risk was also comparable (26% vs 30–39%). The frequency of secondary AML was more common in the VIALE-C study (38%), compared to 25% in VIALE-A and 27% in the ALLG NBCR cohort (*p* = 0.002). The form of non-intensive therapy used for initial treatment of AML in the ALLG NBCR included HMA (67%), LDAC (13%), or other (20%), and the corresponding median bone marrow blast percentages were 26% (>30% blasts in 20% of patients), 62% and 79%, respectively. After a median follow-up time of 32 months (range 10–36) in the ALLG NBCR cohort, median OS for patients treated with either HMA or LDAC was 9.9 and 4.1 months (Fig. 1F), respectively, remarkably similar to the median OS observed for the respective SOC arms of VIALE-A (9.6 months for azacitidine) and VIALE-C (4.1 months for LDAC).

**Table 6:** Characteristics and outcomes of patients enrolled to the VIALE-A, VIALE-C and the ALLG NBCR receiving low-intensity therapy^#^.

Characteristics	VIALE-A (*n* = 286)	AZA-PBO (*n* = 145)	VIALE-C (*n* = 143)	LDAC-PBO (*n* = 68)	NBCR (*n* = 64)
Age, years, median (range)	76 (49–91)	76 (60–90)	76 (36–93)	76 (41–88)	77 (54–92)
Age ≥75 years, *n* (%)	174 (61)	87 (60)	82 (57)	40 (59)	38 (59)
Male gender, *n* (%)	172 (60)	87 (60)	78 (55)	39 (57)	41 (64)
AML type					
- De novo	214 (75)	110 (76)	85 (59)	45 (66)	47 (73)
- Secondary	72 (25)	35 (24)	58 (41)	23 (34)	17 (27)
Secondary AML type					
- Therapy related	26/72 (36)	9/35 (26)	6/58 (10)	4/23 (17)	6/17 (35)
- Prior hematologic disorder	46/72 (64)	26/35 (74)	52/58 (90)	19/23 (83)	11/17 (65)
Cytogenetic risk according to NCCN version 2.2016, *n* (%)					
- Favorable	-	-	1 (1)	3 (5)	-
- Intermediate	182 (64)	89 (61)	90 (65)	43 (65)	40/54 (74)
- Adverse	104 (36)	56 (39)	47 (34)	20 (30)	14/54 (26)
*NPM1* mutation, *n* (%)	27/163 (17)	17/86 (20)	18 (16)	7 (14)	10/25 (40)
*FLT3* mutation, *n* (%)	29/206 (14)	22/108 (20)	20 (18)	9 (17)	5/26 (19)
Treatment response (%)					
- CR/CRi	66.4	28.3	48	13	31.6
- CR	36.7	17.9	27	7	15.8
OS, months, median (95% CI)	14.7 (11.9–18.7)	9.6 (7.4–12.7)	8.4 (5.9–10.1)	4.1 (3.1–8.1)	9.3 (5.3–19.3)

^#^*N* represents the total population; for individual variables, some data were not measured/reported, and the appropriate denominator is specified in these instances. Some percentages do not add up to 100 due to rounding.

*AML* acute myeloid leukemia, *AZA* azacitidine, *CR* complete remission, *CRi* CR with incomplete hematologic recovery, *LDAC* low-dose cytarabine, *NCCN* National Comprehensive Cancer Network, *OS* overall survival, *PBO* placebo.

Among patients receiving low-intensity therapy in the ALLG NBCR, only 5 patients were documented to undergo testing for the *IDH1* mutation, and none of them tested positive. It is worth noting that systematic data capture for *IDH1/2* mutation on the ALLG NBCR only commenced in October 2017. A comparison with the standard arm of the AGILE study [7], which compared ivosidenib or placebo plus azacitidine, was therefore not performed.

## Discussion

The ALLG NBCR was established as an umbrella framework for the collection of SOC AML patient data and for linkage to both sample collection (under an opt-in consent approach) and possible co-enrollment to ALLG clinical trials. Several large population-based AML registries such as the Surveillance, Epidemiology and End Results (SEER) database in United States [14–16], the Swedish Acute Leukemia Registry [17–19], and the Danish National Acute Leukemia Registry [20] have described the longitudinal treatment outcomes of patients with AML over several decades. Key features distinguishing the ALLG NBCR from other cancer registries are source data review of karyotype by an independent cytogeneticist and AML diagnosis by an independent team of hematologists, documentation of each component of treatment and dose received by the patient, and source verification of treatment outcomes using primary bone marrow reports.

In this study, we compared treatment practices and outcomes in real-world patients enrolled to the ALLG NBCR with study populations included as the control arm of several frontline treatment indications (midostaurin, GO, CPX-351, oral azacitidine and venetoclax) prior to their approval and availability in Australia. Overall, we observed that survival outcome among comparable cohorts captured in the ALLG NBCR appeared either similar to, or superior to the control arm of clinical trials examining the benefit of adding midostaurin, GO, CPX-351, CC-486 or venetoclax to standard of care. This was despite substantial variation in patient demographic profile and treatment regimens used in various clinical trials, compared to “real-world” practice.

The intensive chemotherapy backbone used by most centers in Australia has been influenced in part by locally conducted cooperative group clinical trials [21–24] and subsequent modifications [25–27]. These include the use of idarubicin as the dominant anthracycline and delivery of higher-dose cytarabine (1000–3000 mg/m^2^) during induction, especially for patients up to the age of 55. Registration clinical trials for midostaurin, GO and CPX-351 used a backbone comprising daunorubicin 60 mg/m^2^ and cytarabine 100–200 mg/m^2^. The choice of anthracycline (idarubicin versus daunorubicin) has been the subject of several studies and meta-analyses [28–30]. Although there is no general impact of cytotoxic dose intensity on outcome expected with the regimens used in this study, intensified daunorubicin in AML induction (90 mg/m^2^ vs 60 mg/m^2^) has been shown in a post-hoc analysis to provide some benefit to patients with *FLT3*-ITD [31, 32]. In addition, higher-dose cytarabine induction in another *post-hoc* analysis of a randomized trial was shown to improve survival outcomes among patients <46 years [33].

Among patients with *FLT3* mutated AML, treatment backbone differences (e.g. 7 + 3 vs higher-dose cytarabine based induction) did not appear to result in sustained differences in treatment outcome within the ALLG NBCR cohort, nor in numerical comparison to 4-year survival with the control arm of RATIFY (Fig. 1A and Table 2). A higher baseline WBC was noted for patients in the ALLG NBCR cohort, likely reflecting a less restricted patient population than required for entry to clinical trials. In RATIFY, 20% of patients with *FLT3* mutation at screening were not randomized to the study [2]. Despite the higher WBC in the ALLG NBCR population, the 30-day early death rate was ~2%, similar to RATIFY (~4%).

Patients with de novo AML aged 50–70 years in the ALLG NBCR appeared to have complete remission (CR/CRp) and overall survival outcomes comparable to the GO arm of the ALFA-0701 study (Fig. 1B and Table 3). Although GO is associated with a dominant survival benefit among patients with core-binding factor AML [34], the proportion of patients with favorable cytogenetic risk de novo AML aged between 50–70 years was only 9% and 4% in the NBCR and ALFA-0701 cohorts, respectively. One major difference in practice between the NBCR cohort and the control arm of the ALFA-0701 study was the predominant use of idarubicin (36 mg/m^2^) in the NBCR cohort, compared to daunorubicin (180 mg/m^2^) in the ALFA-0701 study. The potential impact of idarubicin in enhancing induction response rates compared to daunorubicin is supported by a prior study from France in older patients with AML that demonstrated the association between idarubicin-based induction and a higher complete remission rate, without significant improvement in overall survival [35].

For secondary/therapy-related AML, patients in the ALLG NBCR appeared to have better outcomes than patients enrolled in the control 7 + 3 arm of the CPX-351 study (Table 4). This included higher rates of CR/CRi (53% vs 33%), as well as longer median OS (9.46 versus 5.95 months). Potential factors confounding interpretation of outcomes in the NBCR cohort include more frequent use of higher-dose cytarabine consolidation and fewer patients ≥70 years in the ALLG NBCR cohort, compared to the control arm of the CPX-351 registrational study. These important differences could partly explain the better-than-expected outcomes for patients in the ALLG NBCR relative to the CPX-351 study. Although landmarked OS outcome post-HCT was similar between the NBCR cohort and the control arm of the CPX-351 study, only 6 patients with secondary/therapy-related AML were transplanted in the NBCR cohort. Therefore, survival outcome in this sub-group should be interpreted with caution.

In the comparison of ALLG NBCR outcomes with patients in the QUAZAR oral azacitidine study, the ALLG NBCR cohort was significantly younger, reflecting a bias in the ALLG NBCR population and perhaps explaining the relatively better-than-expected outcomes for patients in the NBCR cohort, even when prior HCT was excluded (Table 5).

Elderly patients unfit for intensive chemotherapy are underrepresented in the ALLG NBCR. For the first 5 years of the registry, there was preferential enrolment of patients under the age of 65 at tertiary and metropolitan centers which was in keeping with the procedure to enroll to the NBCR for clinical trial inclusion. Therefore, the median age of patients enrolled to the ALLG NBCR was significantly younger (median age 58 years) than those reported by the Australian Institute of Health and Welfare (AIHW, median age 68.5 years) [36]. Before December 2021, azacitidine was the sole approved low-intensity treatment option in Australia, reimbursed for individuals with 20–30% bone marrow blasts. The approval of venetoclax-azacitidine from December 2021 is expected to enhance the inclusion of older patients with AML in the ALLG NBCR.

Overall, although the majority of cohorts in the ALLG NBCR showed similarities to the concordant control arm of randomized studies, important differences in patient demographics, standard therapy and transplantation frequency were noted. Although there is a strong desire to support the concept of using single arm study outcomes compared to synthetic historical control populations to support drug approvals, the complexity and importance of matching study populations to counteract baseline and treatment biases with potential impact on outcome remains an important consideration and a potential hurdle to utilization of real-world data for comparative studies with new agents.

One limitation of the current study was the limited population coverage, compared to legislated cancer registries. The proportion of patients with AML captured in the ALLG NBCR is approximately 30% of cases reported to the national body, the AIHW [36]. The interpretation of outcomes from our registry cohort should be contextualized within a developed country setting, benefiting from free healthcare access and robust supportive care, including intensive care facilities. As a nation, emphasis to data linkages is evolving, and in the future challenges of data collection, completeness and analysis are hoped to be overcome with greater awareness and support to national infrastructure like the ALLG’s NBCR.

When national regulators assess the potential health economic benefits associated with new anti-cancer therapies, the availability of relevant real-world data is often lacking. It this hoped that this analysis of patient outcomes in the ALLG NBCR prior to the introduction of novel therapies highlights the diversity of clinical practice differences in routine clinical care. Such differences are likely to become larger and even more complex after introduction of recently approved drugs for frontline use in AML, such as midostaurin, CPX-351, GO, venetoclax, oral azacitidine and ivosidenib. In conclusion, this study highlights the growing complexity reimbursement agencies will face when trying to evaluate the comparative benefit a new drug for patients with AML using “real-world” data. This situation will be exacerbated further by the many inherent variables that already exist in standard of care practice globally. This report serves to highlight some of these differences in the context of Australian clinical practice prior to incorporation of new drug indications into routine clinical practice that commenced in 2018. Although there is growing desire to use historical datasets to assess the impact of novel therapies, this work underscores the large variations in standard of care practice that likely exist and the need to compile data from multiple historical datasets to ensure that geographic differences in treatment outcomes are accounted for.

### Supplementary information

Supplemental Material

## Data Availability

Data will be made available upon reasonable request. Data requestors will need to sign a data access request and submit a proposal to Prof Andrew Wei to be reviewed and approved by the Australasian Leukaemia and Lymphoma Group (ALLG) Registry Operation Committee.

## References

[1] Wei AH, Tiong IS (2017). Midostaurin, enasidenib, CPX-351, gemtuzumab ozogamicin, and venetoclax bring new hope to AML. Blood.

[2] Stone RM, Mandrekar SJ, Sanford BL, Laumann K, Geyer S, Bloomfield CD (2017). Midostaurin plus chemotherapy for acute myeloid leukemia with a FLT3 mutation. N. Engl J Med.

[3] Castaigne S, Pautas C, Terré C, Raffoux E, Bordessoule D, Bastie J-N (2012). Effect of gemtuzumab ozogamicin on survival of adult patients with de-novo acute myeloid leukaemia (ALFA-0701): a randomised, open-label, phase 3 study. Lancet.

[4] Lambert J, Pautas C, Terre C, Raffoux E, Turlure P, Caillot D (2019). Gemtuzumab ozogamicin for de novo acute myeloid leukemia: final efficacy and safety updates from the open-label, phase III ALFA-0701 trial. Haematologica.

[5] DiNardo C, Jonas B, Pullarkat V, Thirman M, Garcia J, Wei A (2020). Azacitidine and venetoclax in previously untreated acute myeloid leukemia. N. Engl J Med.

[6] Wei AH, Montesinos P, Ivanov V, DiNardo CD, Novak J, Laribi K (2020). Venetoclax plus LDAC for newly diagnosed AML ineligible for intensive chemotherapy: a phase 3 randomized placebo-controlled trial. Blood.

[7] Montesinos P, Recher C, Vives S, Zarzycka E, Wang J, Bertani G (2022). Ivosidenib and azacitidine in IDH1-mutated acute myeloid leukemia. N. Engl J Med.

[8] Lancet JE, Uy GL, Cortes JE, Newell LF, Lin TL, Ritchie EK (2018). CPX-351 (cytarabine and daunorubicin) liposome for injection versus conventional cytarabine plus daunorubicin in older patients with newly diagnosed secondary acute myeloid leukemia. J Clin Oncol.

[9] Wei AH, Dohner H, Pocock C, Montesinos P, Afanasyev B, Dombret H (2020). Oral azacitidine maintenance therapy for acute myeloid leukemia in first remission. N. Engl J Med.

[10] Grimwade D, Hills RK, Moorman AV, Walker H, Chatters S, Goldstone AH (2010). Refinement of cytogenetic classification in acute myeloid leukemia: determination of prognostic significance of rare recurring chromosomal abnormalities among 5876 younger adult patients treated in the United Kingdom Medical Research Council trials. Blood.

[11] Dohner H, Estey EH, Amadori S, Appelbaum FR, Buchner T, Burnett AK (2010). Diagnosis and management of acute myeloid leukemia in adults: recommendations from an international expert panel, on behalf of the European LeukemiaNet. Blood.

[12] Swerdlow SH, Campo E, Harris NL, Jaffe ES, Pileri SA, Stein H, et al. WHO Classification of Tumours of Haematopoietic and Lymphoid Tissues. Lyon: IARC Press; (2008).

[13] Lancet JE, Uy GL, Newell LF, Lin TL, Ritchie EK, Stuart RK (2021). CPX-351 versus 7+3 cytarabine and daunorubicin chemotherapy in older adults with newly diagnosed high-risk or secondary acute myeloid leukaemia: 5-year results of a randomised, open-label, multicentre, phase 3 trial. Lancet Haematol.

[14] Thein MS, Ershler WB, Jemal A, Yates JW, Baer MR (2013). Outcome of older patients with acute myeloid leukemia: an analysis of SEER data over 3 decades. Cancer.

[15] Song X, Peng Y, Wang X, Chen Y, Jin L, Yang T (2018). Incidence, survival, and risk factors for adults with acute myeloid leukemia not otherwise specified and acute myeloid leukemia with recurrent genetic abnormalities: analysis of the surveillance, epidemiology, and end results (SEER) database, 2001–2013. Acta Haematol.

[16] Zeidan AM, Podoltsev NA, Wang X, Bewersdorf JP, Shallis RM, Huntington SF (2019). Temporal patterns and predictors of receiving no active treatment among older patients with acute myeloid leukemia in the United States: a population-level analysis. Cancer.

[17] Juliusson G, Antunovic P, Derolf Å, Lehmann S, Möllgård L, Stockelberg D (2009). Age and acute myeloid leukemia: real world data on decision to treat and outcomes from the Swedish Acute Leukemia Registry. Blood.

[18] Juliusson G, Lazarevic V, Horstedt AS, Hagberg O, Hoglund M, Swedish Acute Leukemia Registry G. (2012). Acute myeloid leukemia in the real world: why population-based registries are needed. Blood.

[19] Juliusson G, Abrahamsson J, Lazarevic V, Antunovic P, Derolf A, Garelius H (2017). Prevalence and characteristics of survivors from acute myeloid leukemia in Sweden. Leukemia.

[20] Ostgard LS, Norgaard JM, Raaschou-Jensen KK, Pedersen RS, Ronnov-Jessen D, Pedersen PT (2016). The Danish national acute leukemia registry. Clin Epidemiol.

[21] Bishop JF, Lowenthal RM, Joshua D, Matthews JP, Todd D, Cobcroft R (1990). Etoposide in acute nonlymphocytic leukemia. Australian Leukemia Study Group. Blood.

[22] Bishop JF, Matthews JP, Young GA, Szer J, Gillett A, Joshua D (1996). A randomized study of high-dose cytarabine in induction in acute myeloid leukemia. Blood.

[23] Bradstock KF, Matthews JP, Lowenthal RM, Baxter H, Catalano J, Brighton T (2005). A randomized trial of high-versus conventional-dose cytarabine in consolidation chemotherapy for adult de novo acute myeloid leukemia in first remission after induction therapy containing high-dose cytarabine. Blood.

[24] Bradstock KF, Link E, Di Iulio J, Szer J, Marlton P, Wei AH (2017). Idarubicin dose escalation during consolidation therapy for adult acute myeloid leukemia. J Clin Oncol.

[25] Low M, Lee D, Coutsouvelis J, Patil S, Opat S, Walker P (2013). High-dose cytarabine (24 g/m2) in combination with idarubicin (HiDAC-3) results in high first-cycle response with limited gastrointestinal toxicity in adult acute myeloid leukaemia. Intern Med J.

[26] Löwenberg B (2013). Sense and nonsense of high-dose cytarabine for acute myeloid leukemia. Blood.

[27] Tiong IS, Fielding K, Fong CY, Bortz H, Avery S, Grigg AP (2017). Comparison of frontline induction therapy using idarubicin in combination with high-dose (HDAC3) versus intermediate-dose cytarabine (IDAC3) in adult acute myeloid leukemia. Blood.

[28] Wang H, Xiao X, Xiao Q, Lu Y, Wu Y (2020). The efficacy and safety of daunorubicin versus idarubicin combined with cytarabine for induction therapy in acute myeloid leukemia: a meta-analysis of randomized clinical trials. Med (Baltim).

[29] Sekine L, Morais VD, Lima KM, Onsten TG, Ziegelmann PK, Ribeiro RA (2015). Conventional and high-dose daunorubicin and idarubicin in acute myeloid leukaemia remission induction treatment: a mixed treatment comparison meta-analysis of 7258 patients. Hematol Oncol.

[30] Tefferi A, Gangat N, Shah M, Alkhateeb H, Patnaik MS, Al-Kali A (2022). Daunorubicin-60 versus daunorubicin-90 versus idarubicin-12 for induction chemotherapy in acute myeloid leukemia: a retrospective analysis of the Mayo Clinic experience. Haematologica.

[31] Burnett AK, Russell NH, Hills RK, Kell J, Cavenagh J, Kjeldsen L (2015). A randomized comparison of daunorubicin 90 mg/m^2^ vs 60 mg/m^2^ in AML induction: results from the UK NCRI AML17 trial in 1206 patients. Blood.

[32] Burnett AK, Russell NH, Hills RK, United Kingdom National Cancer Research Institute Acute Myeloid Leukemia Study G. (2016). Higher daunorubicin exposure benefits FLT3 mutated acute myeloid leukemia. Blood.

[33] Willemze R, Suciu S, Meloni G, Labar B, Marie JP, Halkes CJ (2014). High-dose cytarabine in induction treatment improves the outcome of adult patients younger than age 46 years with acute myeloid leukemia: results of the EORTC-GIMEMA AML-12 trial. J Clin Oncol: Off J Am Soc Clin Oncol.

[34] Hills RK, Castaigne S, Appelbaum FR, Delaunay J, Petersdorf S, Othus M (2014). Addition of gemtuzumab ozogamicin to induction chemotherapy in adult patients with acute myeloid leukaemia: a meta-analysis of individual patient data from randomised controlled trials. Lancet Oncol.

[35] Gardin C, Chevret S, Pautas C, Turlure P, Raffoux E, Thomas X (2013). Superior long-term outcome with idarubicin compared with high-dose daunorubicin in patients with acute myeloid leukemia age 50 years and older. J Clin Oncol.

[36] Australian Institute of Health and Welfare. Cancer data in Australia. Available from: https://www.aihw.gov.au/reports/cancer/cancer-data-in-australia/contents/cancer-summary-data-visualisation. Accessed: 28-Jan- (2022).

