# Supplementary material for: How comparable are patient outcomes in the “real-world” with populations studied in pivotal AML trials?
Source: Blood Cancer J. 2024 Mar 26;14(1):54. doi: 10.1038/s41408-024-00996-x (PMC10965987; doi:10.1038/s41408-024-00996-x)
Supplement: Supplementary file 1 — Supplemental Material [file 41408_2024_996_MOESM1_ESM.docx]

**Supplementary Appendix**

Supplement to Tiong, et al (*Blood Cancer Journal* 2024). “**How comparable are patient outcomes in the “real-world” with populations studied in pivotal AML trials?”**

1. **SUPPLEMENTAL INFORMATION** for the ALLG AMLM15, AMLM16 and AMLM21 trials
2. **SUPPLEMENTAL TABLES**

**Table S1.** Variables recorded on case report forms used by the ALLG National Blood Cancer Registry.

**Table S2.** Summary of data quality for the key data fields from NBCR.

**Table S3**. Summary of patient demographics and disease characteristics in NBCR with diagnosis of AML up to 30-Apr-2018.

1. **SUPPLEMENTAL FIGURES**

**Figure S1.** Patient accrual over time onto Australasian Leukaemia and Lymphoma Group (ALLG) National Blood Cancer Registry (NBCR) with cutoff registration date 30-Apr-2018.

**Figure S2.** Flow diagram of patient selection for key subgroup analyses. *Abbreviations*: AML: acute myeloid leukemia; APL: acute promyelocytic leukemia; CBF: core-binding-factor; CML: chronic myeloid leukemia; CR: complete remission; CRi: complete remission with incomplete hematologic recovery; sAML: secondary AML; tAML: therapy-related AML.

1. **SUPPLEMENTAL INFORMATION**

***A pilot study exploring high-dose lenalidomide maintenance therapy in adult acute myeloid leukemia (AML): AMLM15 study by the Australasian Leukaemia and Lymphoma Group (ALLG)***

Clinical trial registration number: ACTRN12610000627055

Key eligibility criteria: In first complete remission (CR) or CR with incomplete hematologic recovery (CRi) after standard induction and consolidation chemotherapy; lenalidomide therapy must be able to commence within 6–16 weeks since completion of the last dose of consolidation chemotherapy; adequate baseline bone marrow reserve (neutrophils ≥1.0 x 10^9^/L and platelets ≥75 x 10^9^/L) prior to study treatment.

Study therapy: Five study cohorts received two 28-day continuous cycles of “high-dose” lenalidomide (10, 20, 30, 40 or 50 mg daily), followed by up to 10 subsequent cycles of 28-day “low-dose” maintenance lenalidomide (10 mg daily).

This study was presented as an abstract at the American Society of Hematology (ASH) meeting in 2014: Andrew Wei, et al. Maintenance Lenalidomide for Adults Aged 18-65 Years with AML in First Complete Remission after Intensive Chemotherapy: A Phase Ib Dose-Escalation Study of the Australasian Leukemia and Lymphoma Group (ALLG). Blood 2014; 124 (21): 944. <https://doi.org/10.1182/blood.V124.21.944.944>.

***Sorafenib in combination with intensive chemotherapy for previously untreated adult FLT3-ITD positive AML: a phase 2 randomized double-blind placebo controlled multi-center study (ALLG AMLM16).***

Clinical trial registration number: ACTRN12611001112954

Key eligibility criteria: aged 18–65 years with newly diagnosed AML and *FLT3*-ITD allelic ratio ≥0.05.

Study therapy: Patients were randomized 2:1 to induction chemotherapy combined with either sorafenib or placebo. Sorafenib or placebo 400 mg twice daily was administered orally days 4–10 during induction and all consolidation cycles. During maintenance, sorafenib or placebo was administered no sooner than day 42 after commencing the last consolidation cycle, but no later than day 90 if awaiting recovery of neutrophils ≥1.0 x 10^9^/L and platelets ≥75 x 10^9^/L. Maintenance was delivered for up to 12x 28-day cycles.

Published: Loo et al, Sorafenib plus intensive chemotherapy in newly diagnosed FLT3-ITD AML: a randomized, placebo-controlled study by the ALLG. Blood 2023. <https://doi.org/10.1182/blood.2023020301>.

***A phase 1b/II clinical evaluation of ponatinib in combination with 5-azacitidine in patients failing prior therapy for FLT3-ITD positive acute myeloid leukemia (AMLM21)***

Clinical trial registration number: ACTRN12614000810617

Key eligibility criteria: aged ≥18 years with *FLT3*-ITD positive AML who failed prior chemotherapy (≤2 lines) or who were considered unfit for frontline intensive chemotherapy; prior exposure to other FLT3 inhibitors were allowed (need to demonstrate absence of *FLT3*-TKD mutation); no prior exposure to a hypomethylating agent.

Study therapy: At dose level 1, patients received azacitidine 60 mg/m^2^ on days 1–5 and 8–9 and ponatinib 30 mg daily on days 5–25 of each 28-day cycle. In patients not achieving CR or CRi after cycle 1, the ponatinib dose was increased to 45 mg during cycle 2.

This study was presented as an abstract at the American Society Meeting (ASH) meeting in 2021: David Kipp, et al. A Phase-Ib/II Clinical Evaluation of Ponatinib in Combination with Azacitidine in *FLT3*-ITD and *CBL*-Mutant Acute Myeloid Leukemia (PON-AZA study). Blood 2021; 138 (Supplement 1): 2350. doi: <https://doi.org/10.1182/blood-2021-154247>.

1. **SUPPLEMENTAL TABLES**

**Table S1.** Variables recorded on case report forms used by the ALLG National Blood Cancer Registry.

| ***Registration CRF*** |
| --- |
| Date of written consent |
| Patient initials |
| Date of birth |
| Gender |
| Consent to sample collection (optional) |
| Consent to provide healthcare identification number (optional) |
| ***AML diagnosis CRF*** |
| ECOG performance status |
| Date of original AML diagnosis |
| Antecedent hematological disorder |
| Site determined WHO AML classification |
| Medical monitor verified final diagnosis (WHO 2008) |
| Full blood examination – upload report (for review by medical monitor) |
| Bone marrow biopsy – upload report (for review by medical monitor) |
| Flow cytometry – upload report (for review by medical monitor) |
| Molecular genetics – upload report (for review by medical monitor) |
| - *FLT3*-ITD and allelic ratio |
| - *NPM1* mutation |
| - *CEBPA* mutation |
| - *IDH1/2* mutation |
| Cytogenetics – upload report (for review by cytogeneticist) |
| - Monosomal karyotype |
| - Complex karyotype (Dohner 2010) |
| - MRC risk classification (Grimwade 2010) |
| - Chromosome abnormalities |
| ***Treatment & Response CRF*** |
| - Clinical trial participation (Yes/No, specify) |
| - For each cycle of chemotherapy (date, type, regimen, and dose) |
| - Response to treatment (Dohner 2010) |
| - Transplant (date, timepoint, donor, conditioning, T-cell depletion) |
| ***Relapse CRF*** |
| - Bone marrow biopsy – upload report |
| - Cytogenetics – upload report |
| - [Treatment & Response CRF] for further therapy |
| ***Follow Up*** |
| - Survival status (date of death or end of follow up, cause of death) |
| - Disease status |
| - MRD monitoring result |

**Table S2.** Summary of data quality for the key data fields from NBCR.

| **Selected variables** | **% complete** |
| --- | --- |
| Date of diagnosis | 100 |
| FBE – white blood cell count | >99 |
| Bone marrow biopsy report | 94 |
| Flow cytometry report | 93 |
| Molecular genetics among intensively treated cohort (n=799) |  |
| - *FLT3*-ITD | 87 |
| - *FLT3*-ITD allelic ratio | 87 |
| - *FLT3*-TKD | 63 |
| - *NPM1* | 73 |
| - *CEBPA* | 16 |
| - *IDH1/2* | 20 |
| Molecular genetics among non-intensively treated cohort (n=81) |  |
| - *FLT3*-ITD | 49 |
| - *FLT3*-ITD allelic ratio | 49 |
| - *FLT3*-TKD | 35 |
| - *NPM1* | 47 |
| - *CEBPA* | 6 |
| - *IDH1/2* | 22 |
| Cytogenetics report | 93 |
| Site determined AML classification | 100 |
| Medical monitor verified diagnosis | 100 |
| Clinical trial participation | 89 |
| First line treatment |  |
| - Date | 91 |
| - Treatment response | 84 |
| Follow up for surviving patients |  |
| - ≥12 months | 83 |
| - ≥2 years | 63 |
| Cause of death | 89 |

**Table S3**. Summary of patient demographics and disease characteristics in NBCR with diagnosis of AML up to 30-Apr-2018.

| **Characteristics** | **N=942** |
| --- | --- |
| **Age**, median years (range) | 59 (16–92) |
| - Age 60–74 years, n (%) | 377 (40.0) |
| - Age ≥75 years, n (%) | 89 (9.4) |
| **Male gender**, n (%) | 517 (54.9) |
| **White blood cell** (x 10^9^/L), median (IQR) | 7.4 (2.5-34.3) |
| - WBC >40 x 10^9^/L, n (%) | 211 (22.5) |
| - WBC >100 x 10^9^/L, n (%) | 73 (7.8) |
| **Secondary AML,** n (%) | 115 (12.2) |
| - Therapy-related | 51 (5.4) |
| - Antecedent myelodysplastic syndrome | 40 (4.2) |
| - Antecedent chronic myelomonocytic leukemia | 17 (1.8) |
| - Antecedent myeloproliferative neoplasm | 7 (0.7) |
| **MRC 2010 cytogenetic risk**, n (%) |  |
| - Favorable | 95 (10.1) |
| - Intermediate | 591 (62.7) |
| - Adverse | 181 (19.2) |
| - Failed/Unknown | 75 (8.0) |
| **Complex karyotype**, n (%)* | 135 (15.7) |
| **Monosomal karyotype**, n (%) | 104 (12.1) |
| **Genetic mutations**, n (%) |  |
| - *NPM1* | 228 (35.1) |
| - *FLT3*-ITD | 179 (23.3) |
| - *FLT3*-TKD | 32 (6.0) |
| - *CEBPA* biallelic mutation | 9 (6.7) |
| - *IDH1*-R132 | 27 (14.5) |
| - *IDH2*-R140 or R172 | 31 (16.8) |
| **WHO 2008 Classification**, n (%) |  |
| Myelodysplasia-related changes | 267 (28.3) |
| Not otherwise specified | 264 (28.0) |
| Recurrent genetic abnormalities† |  |
| *-* Mutated *NPM1* | 214 (22.7) |
| *-* Mutated *CEBPA* | 12 (1.3) |
| *- CBFB::MYH11* | 53 (5.6) |
| *- RUNX1::RUNX1T1* | 37 (3.9) |
| *-* Rearranged *KMT2A* | 32 (3.4) |
| - Inv(3) or t(3;3) | 5 (0.5) |
| *- DEK::NUP214* | 4 (0.4) |
| Therapy-related myeloid neoplasms | 51 (5.4) |
| Other | 5 (0.5) |
| **Upfront therapy**, n (%) |  |
| - Intensive | 799 (84.8) |
| - Low-intensity | 81 (8.6) |
| - Best supportive care only | 40 (4.2) |
| - Unknown | 22 (2.3) |
| **Allogeneic HCT**, n (%) | 249 (26.4) |
| - In first remission | 198 (21.0) |

* Complex karyotype is defined as per ELN 2017 recommendations.

† Two cases of AML with mutated *NPM1* also had *CEBPA* mutations (one monoallelic and one biallelic). 14 cases with *NPM1* mutation were classified as AML with myelodysplasia-related changes (n=11), therapy-related myeloid neoplasm (n=2), or acute erythroid leukemia (n=1). AML with mutated *CEBPA* included 5 cases with monoallelic mutation as 2008 WHO classification did not distinguish between the two.

*Abbreviations*: AML: acute myeloid leukemia; HCT: hematopoietic cell transplantation; MRC: Medical Research Council; WHO: World Health Organization.

1. **SUPPLEMENTAL FIGURES**

**Figure S1.** Patient accrual over time onto Australasian Leukaemia and Lymphoma Group (ALLG) National Blood Cancer Registry (NBCR) with cutoff registration date 30-Apr-2018.

**Figure S2.** Flow diagram of patient selection for key subgroup analyses. *Abbreviations*: AML: acute myeloid leukemia; APL: acute promyelocytic leukemia; CBF: core-binding-factor; CML: chronic myeloid leukemia; CR: complete remission; CRi: complete remission with incomplete hematologic recovery; sAML: secondary AML; tAML: therapy-related AML.
